# An assessment of appraisals of dating relationship conflicts and perceptions of appropriate coping strategies with psychologically abusive interactions

**DOI:** 10.3389/fpsyg.2023.1286139

**Published:** 2023-12-04

**Authors:** Kimberly Matheson, Daphne Wornovitzky, Jyllenna Landry, Hymie Anisman

**Affiliations:** ^1^Department of Neuroscience, Carleton University, Ottawa, ON, Canada; ^2^Institute of Mental Health Research, University of Ottawa, Ottawa, ON, Canada; ^3^School of Social Work, University of Calgary, Calgary, AB, Canada; ^4^Department of Psychology, University of Saskatchewan, Saskatoon, SK, Canada

**Keywords:** psychological abuse, dating relationships, conflict appraisals, coping, gender, depressive affect

## Abstract

**Introduction:**

Stemming from a stress appraisal and coping perspective, the present investigation developed a methodology for assessing how individuals appraise abusive dating relationship conflicts (Study 1) and the implications of such appraisals for informing coping responses to abusive interactions (Study 2).

**Methods:**

Participants ranging in age from 17 to 29 years (Study 1: 102 males, 339 females; Study 2: 88 males, 362 females) completed a survey in which they were presented with a series of 10 scenarios that conveyed relationship conflict cues that were ostensibly aligned with various forms of psychological abuse.

**Results:**

Factor analyses indicated that blatant actions conducted in privacy were differentiated from more ambiguous public forms of psychological abuse, in that the latter were appraised by both males and females as *more* abusive. Females were further likely to appraise blatant conflicts as more threatening but at the same time more resolvable. Participants who had encountered abuse in their own intimate relationships were especially likely to appraise conflicts as abusive, threatening and uncontrollable. Such appraisals were associated with greater endorsement of avoidant coping strategies in response to an abusive encounter, irrespective of personal relationship experiences.

**Discussion:**

It is suggested that how individuals appraise relationship conflicts may be key to their ability to cope effectively with such encounters or to provide appropriate support to those experiencing psychologically abusive relationships.

## 1 Introduction

Intimate partner violence (IPV) of either a physical, sexual, or psychological nature occurs across cultures, gender, socioeconomic status, and age groups. It was estimated that more than 25% of women 15–49 years of age had experienced physical or sexual abuse at some time and 13% had experienced violence in the preceding year (Sardinha et al., 2022). Psychological abuse was far more common, with 39% of women reporting emotional abuse in the preceding year (Kapiga et al., 2017). Although much of the prevalence rates reflect women’s experiences of abuse, there is increasing evidence that IPV targeting men is a serious problem (Hines, 2015).

Psychological abuse can occur as early as the dating phases of a relationship, including among adolescents (Dardis et al., 2015; Ybarra et al., 2016). It was estimated that 20–50% of college students have experienced an abusive dating relationship (Scherer et al., 2016). Cyber dating abuse (involving a wide range of abusive behaviors through digital interactions) has also been a growing problem (Caridade et al., 2019). The incidence of IPV increased appreciably during the COVID-19 pandemic, including in relationships where it had never been present earlier (Peitzmeier et al., 2022). These statistics are likely underestimates since many cases go unreported to health professionals or legal authorities (United Nations Women, 2022). It is often assumed that the lack of reporting reflects barriers within the legal and health systems that make it difficult for victims to report their experiences. However, there may be cognitive and emotional factors that limit recognition that an individual’s experiences constitute abuse. The present study assessed appraisals of relationship conflicts that included abusive features, and whether such conflict appraisals were associated with experiences of abuse in their own relationships and the particular patterns of coping that individuals thought they would pursue in response to such abuse.

Despite reports of a high incidence of abusive experiences, psychological abuse is often subtle and insidious, making it difficult for victims to identify it for what it is (Spadine et al., 2020). Although definitions vary, psychological abuse may comprise behaviors such as name-calling, lack of affection, social isolation, humiliation, and jealousy, resulting in the target of the abuse feeling threatened (Sackett and Saunders, 1999; Follingstad, 2007). The severity of such abuse often escalates over time, and the point at which “normal” conflicts cross the line to constitute psychological abuse may be ambiguous (Follingstad, 2007; Daw et al., 2023). In effect, dating abuse varies along multiple dimensions that may place different types of strain on the victim. A notable example is its evolution in narcissistic abusive relationships, which often begin with love-bombing and subtle manipulations before victims are subjected to coercive control and gaslighting (Arabi, 2023). In this regard, in some instances, those who are being psychologically victimized may initially be unaware of (or confused about) their predicament (Minto et al., 2021).

Numerous reasons exist for the targets of abuse to be reluctant to define their situations as such, including a motivation to remain in their relationships due to economic factors, the presence of children, how long the relationship had continued, social influences and interactional patterns, contextual characteristics of their situation (demographic, neighborhood, community, and school factors), mental health issues that might create anxiety regarding change, and whether they have safe places to which they can go (Capaldi et al., 2012; Matheson et al., 2020). However, it is possible that targets’ appraisals of their situation reflect cognitive biases in how they interpret abusive behaviors, with implications for how they cope with the conflict and their decisions regarding the maintenance of the relationship (Matheson et al., 2007). In this regard, women who had previously been abused were more likely to subsequently be revictimized in their intimate relationships (Ørke et al., 2018). It was suggested that this might stem from the initial abusive relationship leading to women processing social information differently and counterproductively, essentially misappraising or not recognizing early signals of threats to their well-being (e.g., Zamir et al., 2018). Likewise, adolescents failed to identify subtle warning signs of abuse early in dating relationships (Towler et al., 2020), and even when these acts were overt (e.g., denigration, personal degradation, public degradation, and verbal aggression) they often paid little heed to these or were unaware of actions that ought to be taken (Francis and Pearson, 2021). Appraisals of abusive interactions as benign may contribute to the decision to stay in a relationship, and often it was the negative appraisals of an intimate partner’s behaviors by others (e.g., parents and friends) that were key in an individual’s decision to terminate a psychologically abusive dating relationship (Copp et al., 2015).

Males may be especially reluctant to define aggressive behaviors toward themselves or other men as constituting abuse due to social stigma and gendered stereotypes associated with being a male victim of abuse (Scott-Storey et al., 2023), and hence, are particularly unlikely to seek help (Thomas and Hart, 2023). Although males were likely to regard abusive behaviors toward women as severe, both females and males were less likely to appraise the same behaviors targeting a male victim as serious (Sikström et al., 2021; Sikström and Dahl, 2023), and were less sympathetic toward male victims (Savage et al., 2017; Thomas and Hart, 2023).

Hand-in-hand with victims of abuse not appraising the situation as a threat is the use of ineffective coping strategies (e.g., avoidant coping), and together with earlier abusive encounters and lower self-esteem, such coping methods predicted a propensity for college-aged women to remain in an abusive dating relationship (Edwards et al., 2011). In fact, women who tend to adopt avoidant coping methods, and who are low in anxiety, perceived males as being less aggressive (Russo and Borelli, 2023). Avoidant coping (which might comprise passive withdrawal, wishful thinking, and emotional containment) might buffer them against the negative mood state ordinarily associated with such experiences, but in so doing the likelihood of engaging in actions to change their situation might be diminished. For example, uniquely among women whose relationships were characterized by abuse, avoidant or emotion-focused coping (e.g., rumination and emotional expression) in response to reminder cues of abuse were associated with greater feelings of positive agency. This pattern was not seen in their responses to other stressors (e.g., academic challenges) in which such coping strategies were associated with elevated anxiety (Matheson et al., 2007). Moreover, victims of abuse often present with psychological symptoms (e.g., depression and PTSD) that were tied to the severity of their experiences as well as the adoption of ineffective coping methods (Matheson et al., 2007; Forth et al., 2022). In this regard, regrettably, the depression associated with being in an abusive relationship further hindered their ability to leave (Mazza et al., 2021).

If these obstacles to leaving were not enough, not only are victims frequently disbelieved when they convey their experiences to others, but they may be blamed for their situation (e.g., Eigenberg and Policastro, 2016). Or, based on a “just world” perspective, the attitude is taken that they must have done something to elicit such behaviors (Valor-Segura et al., 2011). In part, this may be attributable to witnesses of abuse being naïve concerning interpersonal violence (Eigenberg and Policastro, 2016). While physical abuse is easily labeled, taken out of context psychological abuse may not be as easily recognized. On the contrary, such behaviors might be viewed as expressions of jealousy, relationship problems, or alcohol/drug misuse (Neal and Edwards, 2017). Such appraisals may align with the everyday social experiences of those who have not encountered psychological abuse. As a result, victims may have difficulty communicating the severity of their situation to others, and those they seek support from may not comprehend the threat (Sikström and Dahl, 2023). Indeed, women who had *not* been in an abusive relationship were more likely to minimize psychological abuse that they witnessed (Hammock et al., 2015; Hall et al., 2023). Thus, perpetrators of psychological violence were deemed less responsible for their actions than those who had engaged in physical abuse (Wilson and Smirles, 2020).

Research assessing how individuals appraise and cope with psychological abuse in dating relationships has focused on victims of abuse, and has less often addressed how third parties view these events (cf. Sikström et al., 2021; Sikström and Dahl, 2023). Moreover, much of this research focuses on variations in interpretations of targets’ own conflict experiences which are confounded by the nature of the conflicts in their relationships. Third-party appraisals may be further influenced by ambiguities in the reports of those targeted by an abusive partner. The present investigation, stemming from a stress appraisal and coping perspective, developed a methodology for assessing how individuals appraise abusive relationship conflicts (Study 1) and the implications for such perceptions for what they regard as appropriate coping responses (Study 2). To this end, participants were presented with a series of scenarios that conveyed relationship conflict cues that were ostensibly aligned with various forms of psychological abuse. It was anticipated that appraisals of different forms of psychological abuse would vary as a function of individuals’ own experiences, and in particular, that those who previously or currently encountered psychological abuse would be more likely to recognize the cues and perceive them to be distressing and as constituting abuse. In a second study that involved a community sample of young people currently in romantic relationships, the association between such appraisals and how they believed they would cope with an abusive conflict was assessed, with the expectation that the coping methods endorsed would vary as a function of their appraisals of the perpetrator’s behaviors, which themselves may be associated with their own relationship experiences. Of particular interest was whether appraisals that minimize the abuse present in conflicts were associated with avoidant coping strategies, especially among those individuals experiencing abuse in their own relationships.

## 2 Study 1

Third-party interpretations of intimate partner behaviors as psychologically abusive were diminished when there was ambiguity in the reports of the target (Sikström and Dahl, 2023). Likewise, observers’ perceptions of abuse were diminished when the behaviors could be understood as being normal or excusable given the context (Sikström and Dahl, 2023). It was the goal of Study 1 to create a measure that could be used as an index of conflict appraisals within psychologically abusive interactions. By presenting scenarios that convey behaviors that are aligned with features of psychological abuse, appraisals associated with characteristics of the perceiver could be assessed. In so doing, the effects of variations in the ambiguity of the target’s narrative in communicating their experiences are diminished. Response patterns to the varying conflict cues in the scenarios were explored, and it was expected that

1) perceivers’ appraisals would vary depending on whether the conflict cues were more ambiguous with respect to perpetrator motives relative to those that were more blatantly hostile;
2) individuals who had experienced psychological abuse would be more likely to appraise conflict behaviors as abusive, particularly if these were ambiguous;
3) females, relative to males, would be more likely to appraise conflict interactions as abusive.

### 2.1 Method

#### 2.1.1 Participants

Male (*n* = 102) and female (*n* = 339) students (age range 17–29 years; *M* = 19.76, SD = 2.23 years) participated in a study on coping with relationship stressors that was advertised on research sign-up boards as well as posters placed in various campus buildings at a Canadian university. Participants who self-defined as currently involved in heterosexual non-marital relationships of 1–36 months (*M* = 16.7 SD = 15.7 months). Of the participants reporting ethnic group status, the majority were Euro-Caucasian (*n* = 327), with the remainder indicating that they were Asian (*n* = 37), South Asian (*n =* 27), Middle Eastern (*n* = 20), Black (*n* = 13), Hispanic (*n* = 8), or Indigenous (*n* = 3).

#### 2.1.2 Procedures

Following contact, participants were given the option of having the survey mailed to them with a return stamped envelope, picking it up on-site, or completing it online through our website. At the outset of the survey, they were provided with an overview of the purpose and completed an informed consent form. They then responded to background measures, appraisals of a series of scenarios depicting relationship conflicts, and a measure of psychological abuse in their current relationship. Upon completion of the survey, participants were debriefed and provided with contact numbers that included counseling services should they experience distress. As an incentive to participate in the study, participants either received course credit or a $10.00 gift certificate. The procedures were approved by the Carleton University Research Ethics Board (REB #05-005).

#### 2.1.3 Measures

##### 2.1.3.1 Profile of psychological abuse

The Profile of Psychological Abuse (Sackett and Saunders, 1999) is a 27-item self-report measure that uniquely assesses various types of psychological abuse from a current partner. Participants rated the frequency of encountering each behavior on a scale ranging from 0 (never) to 7 (daily). The measure taps into five types of psychological abuse, including jealous control (Cronbach’s α = 0.89), ignoring their partner (Cronbach’s α = 0.72), ridicule (Cronbach’s α = 0.76), criticizing their partner (Cronbach’s α = 0.69), and fear of abuse (Cronbach’s α = 0.81). Mean responses to the items comprising each form of psychological abuse were calculated, as was a total scale score (Cronbach’s α = 0.93). Although originally developed to assess different forms of psychological violence in domestic situations, this measure appeared to reliably assess interactions in dating relationships.

##### 2.1.3.2 Appraisals

Participants completed a measure designed for the present study to assess how they appraised various situations involving conflicts that they may encounter in a romantic relationship. Participants were given 10 scenarios that varied in the nature of the conflict presented based on the different forms of abuse reflected in the Profile of Psychological Abuse (Sackett and Saunders, 1999; see Table 1). The scenarios were created and pilot tested with a sample of 10 graduate students who had academic expertise in relationship conflicts, stress and coping processes, and who were of the same age group as the target population. In the pilot test, comments were provided to clarify the meaning of the final scenarios employed, and whether sufficient context was provided for respondents to evaluate the motives underlying the conflict.

**TABLE 1:** Results of principle components analysis of appraisals of the relationship conflict scenarios in Study 1.

	Scenario	Component 1 loadings				Component 2 loadings			
		Serious	Distress	Control	Interpret	Serious	Distress	Control	Interpret
1	Imagine that you get home from class or work and there’s a phone message from your partner. S/he sounds very irritated and upset, wanting you to call back immediately when you get home.	**0.69**	**0.54**	**0.65**	**0.54**	−0.05	0.06	0.04	−0.01
2	You and your partner go out to dinner with some friends. In a serious conversation, s/he starts making fun of what you have to say, making it clear to everyone that s/he thinks you don’t know anything about the topic.	0.21	0.31	**0.44**	0.16	**0.54**	**0.45**	**0.47**	**0.64**
3	You have a friend visiting from out of town and you inform your partner that you won’t be able to spend much time together for a couple of days. Your partner expresses concern that your friends are taking up a lot of your time, and that you haven’t seen each other much lately.	**0.66**	**0.62**	**0.62**	0.30	0.14	0.07	0.12	0.21
4	You overhear a discussion about a party that your friends went to last week; you discover that your partner was at the party, but you’re only hearing about it now.	**0.53**	**0.70**	**0.40**	**0.71**	0.08	0.14	**0.54**	0.15
5	You go out with your partner and some of his or her friends. But the whole time, he or she seems distracted, in a bad mood, and essentially ignores you.	**0.60**	**0.56**	**0.67**	**0.60**	0.37	0.38	0.16	0.22
6	You are having a disagreement with your partner over some relationship issues and after stating your views on the situation, s/he gets frustrated and stomps out of the house.	**0.63**	**0.47**	**0.61**	**0.44**	0.**42**	0.31	0.24	**0.40**
7	One of your friends calls you to tell you that they saw your boyfriend/girlfriend out with another person on the weekend.	**0.40**	**0.65**	0.34	**0.73**	0.05	0.10	**0.57**	−0.07
8	You came home really late from working at the library. When you walked in your partner was watching TV. You asked what s/he was watching, and s/he turned and slapped you hard in the face.	0.12	0.04	−0.08	0.05	**0.70**	**0.78**	**0.76**	**0.49**
9	You’re out to dinner at a restaurant with your partner, and s/he tells you that you need to cut down on the amount of food you’re eating.	0.02	−0.03	0.09	0.22	**0.73**	**0.68**	**0.72**	**0.55**
10	You have an important paper that you’ve been working hard at for weeks that is due today. You’ve asked your partner to help you out by proof-reading. After s/he has read a few pages, s/he tells you that this is one of the worst essays s/he’s ever seen.	0.15	0.21	0.29	−0.10	**0.63**	**0.63**	**0.56**	**0.67**
	% variance explained	29.8	30.6	34.1	25.1	12.0	12.4	11.3	12.4
	Eigenvalue*	2.98	3.06	3.41	2.51	1.20	1.24	1.14	1.24
	Cronbach’s α	0.68	0.69	0.70	0.63	0.60	0.61	0.65	0.45

*Mean Monte Carlo estimates were 1.24 and 1.17, respectively, for each component. Bold values reflect the items used in the scoring of each subscale.

Following each scenario, participants responded to four rating dimensions that ranged from 1 (not at all) to 5 (extremely) including (1) how serious the participant perceived the situation to be, (2) how distressing it would be to experience, (3) how much control they believed they would have over its resolution, and (4) a set of interpretations or expectations of the outcome of each scenario. With respect to these latter ratings, respondents selected from among five behavioral options how they would interpret the perpetrator’s behavior in the scenario. For example, when asked about the scenario in which the partner gets frustrated and stomps out of the house, respondents could interpret the behavior as relatively benign (e.g., “His or her anger over this argument was kind of excessive. S/he is probably upset about something else.”) to more abusive (e.g., “My partner’s really mad at me, and I’m worried about what will happen when s/he comes back.”). The rankings of these interpretations as constituting psychological abuse were based on pilot participants’ ratings of a prepared list of interpretations of the actions in each scenario in terms of whether they reflected those of a partner who was psychologically abusive.

A series of principal components analyses was conducted based on each set of appraisal ratings employed in the full study sample. To determine the number of components to extract, several criteria were considered including eigenvalues greater than 1, whether the proportion of variance accounted for was greater than 10%, the plateau of the scree plot, and a parallel analysis comparing derived eigenvalues to randomly generated eigenvalues (Monte Carlo simulation; Patil et al., 2008). While there was relative consistency across the four appraisal dimensions, these criteria produced different decisions with the eigenvalues and variance accounted for pointing to three components, whereas the scree plot and parallel analysis suggested two. Examination of the loadings for a three-factor solution (with oblique rotation) demonstrated a lack of consistency in terms of the items that constituted the third factor. As a result, the decision was made to extract two components. Based on rotated (varimax) factor loadings greater than 0.40 (see Table 1), the first component appeared to comprise six scenarios in which the abuse was relatively ambiguous and the conflict was more likely to involve other people, whereas the second component comprised three scenarios in which abuse was more private and blatant. For all the rating dimensions, one scenario (#2) loaded moderately on both components as it was a relatively explicit social humiliation; given the blatant nature of the actions (public ridicule), we chose to include responses to this scenario in scores appraising the blatant abusive interactions. Interitem reliabilities for the first component were satisfactory (Table 1). They were less strong for the second component, but there was no specific item that, if removed, substantially affected the item-total correlations. The small number of items contributing to the second component and the variation in the nature of interactions portrayed likely resulted in the response heterogeneity reflected in the lower interitem reliabilities.

### 2.2 Results and discussion

A series of 2 (Gender: male vs. female) × 2 (Conflict type: ambiguous vs. blatant) mixed measures analyses of variance (ANOVAs) was conducted on the four appraisal ratings. As expected, women appraised the conflicts as more serious, η^2^ = 0.092, *F*(1,439) = 44.29, *p* < 0.001, and distressing, η^2^= 0.157, *F*(1,439) = 82.06, *p* < 0.001 than did men (see Table 2). However, significant Gender × Conflict Type interactions were evident for perceptions of the seriousness of the conflict, η^2^= 0.073, *F*(1,439) = 34.65, *p* < 0.001, and the distress that they would feel, η^2^= 0.085, *F*(1,439) = 40.91, *p* < 0.001. As seen in Table 2, unlike men, women were more likely to regard the blatantly abusive situations as more serious and distressing. Both men and women were equally likely to see blatant conflicts as more controllable than those that were more ambiguous, η^2^= 0.086, *F*(1,439) = 41.53, *p* < 0.001. It is possible that because it is easier to identify the blatant behaviors as aggressive, they are perceived as more readily resolved in whatever manner the target chooses, as perceptions of control are commonly associated with a greater ability to effectively problem-solve.

**TABLE 2:** Mean appraisal ratings (SD) as a function of the nature of the conflict and participant gender in Study 1.

	Male (*n* = 102)		Female (*n* = 339)	
	Ambiguous	Blatant	Ambiguous	Blatant
Seriousness	3.42 (0.72)	3.40 (0.77)	3.61 (0.67)^a^	4.07 (0.64)^b^
Distress	3.20 (0.70)	3.24 (0.73)	3.50 (0.69)^a^	4.08 (0.63)^b^
Control	3.20 (0.60)	3.41 (0.78)	3.38 (0.62)	3.66 (0.78)
Interpret as abuse	2.02 (0.65) ^a^	1.84 (0.59)^b^	1.91 (0.64)^a^	1.52 (0.51)^c^

Columns with different superscripts reflect significantly different simple effects comparisons at *p* < 0.01.

However, on the whole, participants were disinclined to interpret any of the conflict behaviors as abusive, especially in the situations that appeared to be most blatant, η^2^= 0.113, *F*(1,439) = 55.73, *p* < 0.001, although this too was moderated by gender, η^2^= 0.017, *F*(1,439) = 7.50, *p* = 0.017, in that women appeared to be especially unwilling to appraise blatant conflicts as abusive. This pattern of responses among females was counter intuitive as we expected that blatantly abusive actions would be “easiest” to call out for what they were. It might be that the private (one-to-one) element of most of the blatant conflicts contributed to women’s perceptions that such situations were easier to resolve, while at the same time recognizing that the behaviors were serious. Women’s disinclination to view such conflict behaviors as abusive might also reflect an avoidant coping strategy that excuses the perpetrator’s actions as contained in scope and context. Indeed, it has been suggested that young women frequently fail to identify warning signs of abuse early in dating relationships (Towler et al., 2020), and hence do not take actions to preclude further abuse (Francis and Pearson, 2021). Such a possibility aligns with previous reports that by not interpreting such experiences as abusive, women may diminish their distress (Matheson et al., 2007).

Patterns of correlations among appraisals were examined to gain further insights into the co-occurrence of appraisal propensities (Table 3). Not surprisingly, perceptions of the seriousness of the conflict and distress were extremely highly correlated and might best be combined as an index of threat appraisals in this situation. In line with previous research, interpretations of abuse were associated with greater distress and reduced control over the resolution of ambiguous conflicts (or conversely, distress was diminished with lower perceptions of abuse). In contrast, interpreting the blatant scenarios as abusive was associated with lower distress and control appraisals, which might reflect the motivation among women to avoid defining blatant situations as abusive. This said, it is unclear why the difference in the patterns of correlations, but might possibly be due to the more public nature of the ambiguous interactions. We have to be cautious in our speculations regarding the meaning of these relationships, given the correlational nature of these data, as the contributing factors appear to be complex. The direction of these relations was not moderated by gender, despite the finding that women were less likely to appraise the blatant conflicts as constituting abuse.

**TABLE 3:** Pearson correlations among experience of psychological abuse and appraisals of the relationship conflict scenarios (*N* = 441) in Study 1.

	Ambiguous				Blatant			
	Serious	Distress	Control	Interpret	Serious	Distress	Control	Interpret
**Ambiguous conflict scenarios**								
Serious	–							
Distress	0.83***	–						
Control	0.28***	0.22***	–					
Interpret	0.24***	0.28***	-0.16***	–				
**Blatant conflict scenarios**								
Serious	0.42***	0.39***	0.17***	0.01	–			
Distress	0.36***	0.43***	0.15***	0.06	0.90***	–		
Control	0.08	0.06	0.54***	-0.18***	0.14**	0.12**	–	
Interpret	-0.00	-0.01	-0.25***	0.33***	-0.24***	-0.20**	-0.28***	–
**Personal experiences of psychological abuse**								
Jealous control	0.05	0.06	-0.10*	0.37***	-0.06	-0.07	-0.02	0.20***
Ignores	0.14**	0.14**	-0.12**	0.38***	0.05	0.06	-0.17***	0.30***
Ridicules	0.09	0.10*	-0.17***	0.37***	-0.02	-0.03	-0.13**	0.35***
Criticizes	0.08	0.08	-0.10*	0.24***	-0.06	-0.08	-0.05	0.26***
Fear of abuse	0.15***	0.17***	-0.19***	0.42***	-0.04	-0.04	-0.14**	0.34***
Total abuse	0.12*	0.13**	-0.16***	0.46***	-0.03	-0.04	-0.11*	0.34***

*p < 0.05,

**p < 0.01,

***p < 0.001.

Pearson correlations were examined to determine whether there were variations in appraisals as a function of participants’ experiences of psychological abuse in their own dating relationships (Table 3). As expected, personal experiences of abuse (especially when their partner ignored their needs or they lived in fear of abuse) were related to appraisals of ambiguous conflicts as more serious and distressing and less controllable. Irrespective of the nature of the conflict, experiences of abuse in their own relationships were consistently related to whether participants interpreted the behaviors in the conflict scenario to constitute abuse, as well as being less likely to perceive a resolution to the situation as controllable. Thus, it seems that those participants who have more frequently experienced abuse did not blunt (or avoid) recognition of the presence of abuse and the accompanying distress, but rather were more sensitized to situations that could be appraised as abusive, particularly when the motivations of the actions involved were ambiguous. This raises the question of whether such appraisals had implications for the coping mechanisms that contributed to their continued involvement in an abusive situation. These correlations were not moderated by gender, with the exception of perception of control over the resolution of ambiguous conflicts, *F*(1,437) = 7.60, *p* = 0.006. Specifically, whereas experiences of personal abuse were not related to such perceptions in males, *r* = 0.06, *p* = 0.55, among females more frequent abusive experiences were associated with lower perceived control, *r* = −0.22, *p* < 0.001.

## 3 Study 2

Appraisals concerning the threat and controllability of stressors may have considerable sway in determining the coping strategies that individuals endorse, which can, in turn, have behavioral and psychological ramifications (Lazarus and Folkman, 1984). In this regard, appraisals of situations of abuse may have implications for how individuals cope with their relationship conflicts and their well-being (Matheson et al., 2007). In Study 1, appraisals varied as a function of the explicitness of the abuse, ranging from ambiguous behaviors that were enacted in the presence of others to clear aggression in private. Contrary to expectations, the more blatant forms were regarded as less abusive, especially by females. It was suggested that this might reflect a motivation to avoid having to acknowledge that the situation was one that should be treated as abusive.

Understanding how relationship conflicts are appraised has implications for gaining insights into the strategies used to contend with the emotions elicited and the dynamics of the relationship itself (Matheson et al., 2007). Given that those in abusive dating relationships often make efforts to maintain the relationship, there appears to be a potential disjunction between the coping strategies used and their longer-term well-being. As alluded to earlier, when encountering stressors, numerous methods of coping may be used, which generally fall into three broad classes comprising problem-focused, emotion-focused and avoidant coping, although some coping methods (e.g., social support) can serve multiple functions. Typically, when an individual perceives a situation as controllable, they endorse a problem-focused strategy (attempting to diminish or eliminate stressors or contending with them in a thoughtful systematic way) to limit the occurrence of negative psychological outcomes. At the same time, if individuals are motivated to perceive control over a situation, they may be inclined to overestimate their capacity to exert control, which could render them at elevated risk for further adverse effects. Problem-focused coping strategies may also fall short when there is no problem to be solved, for example, in coping with the death of a loved one.

Coping strategies that entail an undue focus on emotions (e.g., rumination, emotional venting, and self-blame) are often maladaptive, but in some situations, this could reflect an effective way of dealing with stressors. In emotionally charged situations, emotion-focused coping (through verbal and non-verbal messages conveying their emotions) lets others know that help is needed, and this approach may facilitate the individual’s ability to come to terms with their feelings, which could potentially reduce distress (Austenfeld and Stanton, 2004). Of course, failure to come to an understanding of these emotions can be counterproductive, most certainly when it entails negative rumination (Nolen-Hoeksema, 2000).

In certain situations, avoidant coping methods (e.g., using avoidance/denial) might be an effective coping strategy in the short run. If nothing else, it may provide temporary relief from an ongoing stressor, perhaps even allowing individuals the opportunity to adopt more effective strategies. Obviously, however, if the stressor persists, then avoidance coping is likely to have negative repercussions that may well escalate. For instance, as we noted in Study 1, despite the fact that blatant abuse ought to be easier to recognize, the disinclination to appraise such interactions as abusive may reflect an avoidant coping strategy that participants might use in their own relationships, and may reduce the likelihood of taking actions to address such behaviors.

Psychological abuse in intimate dating relationships was frequently found to be associated with depressive disorders and the adoption of emotion-focused and avoidant coping (Matheson et al., 2007; Shepherd-McMullen et al., 2015; Gilbert et al., 2023). While instances of depression may occur as a result of the abuse, it is possible that the presence of depressive symptoms influences appraisal and coping processes that allow for the continuation of abuse. Depressive symptoms are associated with less perceived control and more negative cognitive biases that might contribute to threat appraisals (Matheson and Anisman, 2003). Thus, depressive symptoms might contribute to individual differences in appraisals of relationship conflicts. It was important to determine whether the associations between appraisals and abusive experiences reflected a cognitive bias that rendered individuals more sensitive to cues of abusive motives, or whether such appraisals represent a generalized negative bias that is often associated with depression.

Study 2 assessed

1. the replicability of the results of Study 1 in a community sample;
2. the relationship between appraisals of relationship conflicts and beliefs about appropriate coping strategies to contend with abuse;
3. whether the relationships among personal experiences of abuse, appraisals, and coping could be accounted for by the presence of depressive symptoms.

### 3.1 Method

#### 3.1.1 Participants

A community sample of male (*n* = 88) and female (*n* = 362) participants (age range 17–29 years; *M* = 23.23, SD = 3.19 years) completed an online study on coping with relationship stressors that was advertised in community flyers in two major Canadian cities, bulletin boards, as well as notices and links to the study on various websites. Participants were restricted to those who were 29 years of age or less and self-reported as currently involved in heterosexual non-marital relationships of 1–120 months (*M* = 17.1, SD = 12.8 months). Of the participants reporting ethnocultural group status, the majority were Euro-Caucasian (*n* = 307), with the remainder indicating that they were Asian (*n* = 84), South Asian (*n =* 18), Middle Eastern (*n* = 6), Black (*n* = 4), Hispanic (*n* = 3), or Indigenous (*n* = 13).

#### 3.1.2 Procedures

Following an overview of the study procedures, participants provided informed consent. They then responded to an online, mailed or in-person survey, which included measures of psychological abuse in their current relationship, appraisals of relationship conflicts, coping responses to a relationship conflict scenario, and depressive symptoms. Upon completion of the survey, participants were debriefed and provided with contact numbers that included counseling services should they experience distress. As an incentive to participate in the study, participants received a $10.00 gift certificate. The procedures were approved by the Carleton University Research Ethics Board (REB #06-068).

#### 3.1.3 Measures

##### 3.1.3.1 Profile of psychological abuse

The five types of psychological abuse, including jealous control, ignoring their partner, ridicule, criticizing their partner, and fear of abuse were examined to assess replication of patterns with Study 1. Hypothesis testing was conducted on the average total psychological abuse scores (Cronbach’s α0.94).

##### 3.1.3.2 Appraisals

Participants rated their appraisals of the 10 relationship conflict scenarios along the four rating scales used in Study 1. A series of principal components analyses was conducted based on each set of appraisal ratings to assess whether the factor structure replicated (see Table 4). Extraction of two components with a varimax rotation confirmed an identical structure as in Study 1. Interitem reliabilities for the first component were satisfactory (Table 4). They were less strong for the second component, but once again, there was no specific item that, if removed, substantially affected the item-total correlations. The pattern of correlations among the appraisal dimensions for the two types of conflicts replicated those of Study 1 (Table 5). As the perceived seriousness of the conflict and distress were again extremely highly correlated, for all further analyses responses to these appraisal dimensions were averaged to reflect threat appraisals.

**TABLE 4:** Results of principle components analysis of appraisals of the relationship conflict scenarios in Study 2.

	Scenario	Component 1 loadings				Component 2 loadings			
		Serious	Distress	Control	Interpret	Serious	Distress	Control	Interpret
1	Imagine that you get home from class or work and there’s a phone message from your partner. S/he sounds very irritated and upset, wanting you to call back immediately when you get home.	**0.72**	**0.65**	**0.65**	**0.42**	0.03	0.17	0.06	**0.48**
2	You and your partner go out to dinner with some friends. In a serious conversation, s/he starts making fun of what you have to say, making it clear to everyone that s/he thinks you don’t know anything about the topic.	0.37	**0.48**	**0.43**	0.01	**0.54**	**0.45**	**0.42**	**0.67**
3	You have a friend visiting from out of town and you inform your partner that you won’t be able to spend much time together for a couple of days. Your partner expresses concern that your friends are taking up a lot of your time, and that you haven’t seen each other much lately.	**0.67**	**0.67**	0.27	0.32	0.13	0.07	**0.47**	0.33
4	You overhear a discussion about a party that your friends went to last week; you discover that your partner was at the party, but you’re only hearing about it now.	**0.49**	**0.70**	**0.71**	**0.78**	**0.48**	0.14	0.24	0.10
5	You go out with your partner and some of his or her friends. But the whole time, he or she seems distracted, in a bad mood, and essentially ignores you.	**0.70**	**0.65**	**0.76**	**0.74**	0.35	0.38	0.23	0.09
6	You are having a disagreement with your partner over some relationship issues and after stating your views on the situation, s/he gets frustrated and stomps out of the house.	**0.69**	**0.54**	**0.54**	**0.55**	0.18	0.31	0.37	0.30
7	One of your friends calls you to tell you that they saw your boyfriend/girlfriend out with another person on the weekend.	**0.40**	**0.63**	**0.65**	**0.73**	0.37	0.10	0.27	0.12
8	You came home really late from working at the library. When you walked in your partner was watching TV. You asked what s/he was watching, and s/he turned and slapped you hard in the face.	0.11	0.03	0.18	0.15	**0.63**	**0.78**	**0.73**	**0.66**
9	You’re out to dinner at a restaurant with your partner, and s/he tells you that you need to cut down on the amount of food you’re eating.	-0.02	0.15	0.14	0.11	**0.75**	**0.68**	**0.82**	**0.64**
10	You have an important paper that you’ve been working hard at for weeks that is due today. You’ve asked your partner to help you out by proof-reading. After s/he has read a few pages, s/he tells you that this is one of the worst essays s/he’s ever seen.	0.25	0.30	0.24	0.25	**0.54**	**0.63**	**0.74**	**0.53**
	% variance explained	35.8	37.1	40.2	32.3	10.4	10.7	10.3	11.8
	Eigenvalue^1^	3.58	3.71	4.02	3.23	1.05	1.07	1.03	1.18
	Cronbach’s α^2^	0.76	0.76	0.76	0.72	0.59	0.64	0.73	0.58

^1^Mean Monte Carlo estimates were 1.23 and 1.16, respectively for each component.

^2^Cronbach’s αs for combined seriousness/distress to reflect threat were 0.88 (Component 1) and 0.82 (Component 2). Bold values reflect the items used in the scoring of each subscale.

**TABLE 5:** Pearson correlations among appraisals of the conflict scenarios in Study 2 (*N* = 448).

	Ambiguous				Blatant		
	Serious	Distress	Control	Interpret	Serious	Distress	Control
**Ambiguous conflict scenarios**							
Serious	–						
Distress	0.89***	–					
Control	0.11*	0.09	–				
Interpret	0.29***	0.36***	-0.28***	–			
**Blatant conflict scenarios**							
Serious	0.53***	0.50***	0.14**	0.08	–		
Distress	0.49***	0.56***	0.13**	0.12**	0.91***	–	
Control	-0.01	-0.05	0.62***	-0.26***	0.16*	0.13**	–
Interpret	0.00	0.06	-0.31***	0.46***	-0.29***	-0.24**	-0.34***

*p < 0.05,

**p < 0.01,

***p < 0.001.

##### 3.1.3.3 Coping responses

As not all participants experienced abuse in their dating relationships, their coping responses to their own conflicts would likely vary simply due to the nature of the conflicts they encountered. To assess variations in how they believed they would cope with an abusive conflict, participants were provided with a brief paragraph describing a conflict within a relationship and asked to imagine themselves in the situation. The scenario described a person coming home to their partner after a meeting with their boss that went poorly, whereupon the partner behaved unilaterally in a manner that could be construed as psychologically abusive, including criticizing the target’s behavior (e.g., “just what I want to see is you moping around”), ridiculing his or her intelligence and appearance (e.g., “Face it, the problem is you’re not that smart to begin with…. If you look at all competent, it would be a miracle”), and ignoring his or her needs (e.g., “You’re not the only one who’s had a hard day”). At the end of the scenario, the partner stomps out slamming the door.

Participants were asked to imagine that the scenario provided had happened between themselves and their current partner. After they read the scenario, they completed the Survey of Coping Profiles Endorsed (SCOPE; Matheson and Anisman, 2003) that included 50 items to assess 12 coping patterns of coping with stressors, including problem-solving, cognitive restructuring, cognitive avoidance, active distraction, rumination, humor, social support seeking, emotional expression, other- and self-blame, emotional containment, passive resignation, and wishful thinking. Responses were measured on a 5-point scale from 0 (never) and 4 (almost always) to indicate whether participants would use this coping mechanism if they were in the scenario.

A principle components analysis was conducted on the 12 coping subscale scores, and all four decision-making criteria (eigenvalues, variance accounted for, scree plot, and parallel analysis) suggested that they comprised three superordinate ways of coping with the relationship conflict (accounting for 61.9% of the total variance). Based on rotated factor loadings (varimax) greater than 0.50, the first component appeared to reflect five emotional avoidance coping responses (other-blame, self-blame, emotional containment, passive resignation, and wishful thinking) (Cronbach’s α = 0.82). The second component appeared to constitute a problem-focused orientation (problem-solving, cognitive restructuring, active distraction, cognitive avoidance, and humor) (Cronbach’s α = 0.72), whereas the third factor comprised emotional approach coping responses including rumination, social support seeking, and emotional expression (Cronbach’s α = 0.77).

##### 3.1.3.4 Depressive affect

The 21-item Beck Depression Inventory (BDI; Beck et al., 1961) assessed depressive affect. Participants selected from sets of response choices that reflected increasing degrees of depressive severity (e.g., from 0, “I do not feel sad” to 3, “I am so sad or unhappy that I can’t stand it”). Responses were summed to provide an index of depressive affect (Cronbach’s α = 0.92). Scores could range from 0 to 63.

#### 3.1.4 Statistical analyses

Partial correlations among personal experiences of psychological abuse and appraisals of relationship conflicts controlling the severity of depressive affect were examined, followed by a mediation analysis to assess whether appraisals of abusive interactions mediated the relations between personal experiences of abuse and coping strategies that tended to be endorsed in response to an abusive conflict (controlling for depressive affect). The PROCESS macro applying model 4 (Hayes, 2022) was used with bootstrapping procedures using 5,000 resamples to establish the 95% confidence intervals (CI) to assess significance. Where there were significant interactions between personal experiences of psychological abuse in a dating relationship and the appraisal mediators, follow-up analyses were conducted to explore simple effects (PROCESS model 4). For each analysis, the power to detect a medium effect size of ρ = 0.30 at *p* = 0.05 with the sample size of the present study was β = 0.99.

### 3.2 Results and discussion

#### 3.2.1 Replication analyses

A two (Gender: male vs. female) × 2 (Conflict type: ambiguous vs. blatant) mixed measures ANOVA conducted on appraisals of threat indicated, as in Study 1, a significant Gender × Conflict Type interaction, η^2^= 0.034, *F*(1,448) = 15.78, *p* < 0.001. Although men did not see the conflict types to be differentially threatening (*M* = 3.49, SE = 0.068), women regarded the blatantly abusive situations as more threatening (*M* = 4.03, SE = 0.037) than conflicts that were more ambiguous (*M* = 3.63, SE = 0.04). Despite appraising the blatant conflicts as more threatening, once again, women were more likely to regard the resolution of these conflicts as controllable (*M* = 3.57, SE = 0.047) compared to those that were ambiguous (*M* = 3.26, SE = 0.036). In contrast, men were equally likely to see blatant and ambiguous conflicts as moderately controllable (*M* = 3.31, SE = 0.075), η^2^= 0.011, *F*(1,448) = 4.79, *p* = 0.029.

As in Study 1, participants were disinclined to interpret the actor’s conflict behaviors as abusive, especially in the situations that appeared to be blatant (*M* = 1.68, SE = 0.075) compared to ambiguous (*M* = 2.13, SE = 0.075), η^2^= 0.131, *F*(1,448) = 67.69, *p* < 0.001. This too, however, was moderated by gender, η^2^= 0.029, *F*(1,448) = 13.61, *p* < 0.001, in that women appeared to be especially unwilling to appraise blatant (*M* = 1.63, SE = 0.034) rather than ambiguous (*M* = 2.13, SE = 0.004) conflicts as abusive; this was also true of males, but the magnitude of the difference was much less pronounced (blatant *M* = 1.92, SE = 0.068; ambiguous *M* = 2.11, SE = 0.081). Thus, it appears that the patterns of appraisals of relationship conflicts associated with gender and the nature of the conflict replicated in this community sample of young people.

#### 3.2.2 Correlational analyses

As expected, experiences of psychological abuse in their own dating relationships were related to how participants appraised the relationship conflict scenarios, in particular perceptions of the resolution being beyond control and the extent to which actions were interpreted as abusive (Table 6). When controlling for depressive affect, the relations between personal experiences and conflict appraisals were attenuated but largely remained significant. The exception was threat appraisals, wherein after controlling depressive affect, appraisals of ambiguous interactions as threatening were no longer related to personal abuse experiences; perceived threat associated with blatantly abusive interactions and personal abuse experiences became negative (suggesting that depressive affect served as a suppressor variable). It seems that while depressive affect might contribute to the distress individuals who have experienced abuse report in response to relationship conflicts, it does not account for their perceived inability to resolve the situation or their sensitivity to abuse cues.

**TABLE 6:** Correlations between appraisals of relationship conflicts, experiences of psychological abuse, coping in response to an abusive conflict, and depressive affect in Study 2 (*N* = 448).

	Ambiguous			Blatant		
	Threat	Control	Interpret	Threat	Control	Interpret
Psychological abuse	0.14**	-0.25***	0.60***	-0.04	-0.22***	0.57***
Depressive affect	0.19***	-0.24***	0.49***	0.15**	-0.20***	0.33***
Abuse controlling affect	0.04	-0.15**	0.43***	-0.14**	-0.14**	0.48***
**Coping**							
Problem-solving	0.07	0.15**	-0.05	0.10*	0.12*	-0.03
Emotional approach	0.32***	0.03	0.06	0.34***	0.02	-0.07
Emotional avoidant	0.24***	-0.13**	0.41***	0.11*	-0.20***	0.31***

*p < 0.05,

**p < 0.01,

***p < 0.001.

Appraisals of the relationship conflict scenarios were largely unrelated to the endorsement of problem-focused coping efforts in response to an abusive interaction, with the exception that, not surprisingly, such coping efforts were more likely to be endorsed among participants who perceived some degree of control over the resolution of relationship conflicts. While emotional approach coping (rumination, social support seeking, and emotional expression) was solely related to greater threat appraisals, all three appraisal dimensions (threat, control over resolution, and interpretation as abusive) were most strongly associated with emotional avoidance coping strategies (blame, containment, passivity, and withdrawal), mirroring the pattern of associations with depressive affect. It seems possible that appraisals of relationship conflicts might be a part of the constellation of responses that underpin the propensity of individuals who are experiencing abuse to engage in avoidant coping behaviors.

The length of time participants had been in a dating relationship was not significantly related to appraisals, strategies for coping with a conflict, abusive experiences in their own relationships, nor depressive symptoms. Consistent with previous research (Matheson and Anisman, 2003), women (*M* = 2.50, SD = 0.80) were more likely to endorse emotional approach strategies for coping with the relationship conflict than were men (*M* = 2.01, SD = 0.68), η^2^= 0.054, *F*(1,401) = 22.97, *p* < 0.001. There were no gender differences in the endorsement of problem-focused or avoidant coping strategies.

#### 3.2.3 Mediation analyses

A series of mediation analyses was conducted to assess whether the relations between personal experiences of abuse (controlling depressive affect) and the nature of the coping behaviors participants endorsed were mediated by how relationship conflicts were appraised. Although appraisals of control over the resolution of the conflict scenarios were lower among participants with abusive experiences, and such appraisals were positively related to endorsing problem-solving approaches to a relationship conflict (Table 6), the mediated model was not significant, total mediation effect = −0.06, SE = 0.04, CI_.95_ [−0.13, 0.02]. Likewise, although greater threat appraisals were associated with emotional approach forms of coping, such appraisals did not mediate the significant relation between personal experiences of abuse and the lower likelihood of using emotional coping strategies (*B* = −0.18, SE = 0.06, *p* = 0.007), total mediation effect = −0.07, SE = 0.05, CI_.95_ [−0.17,0.02].

**FIGURE 1:**
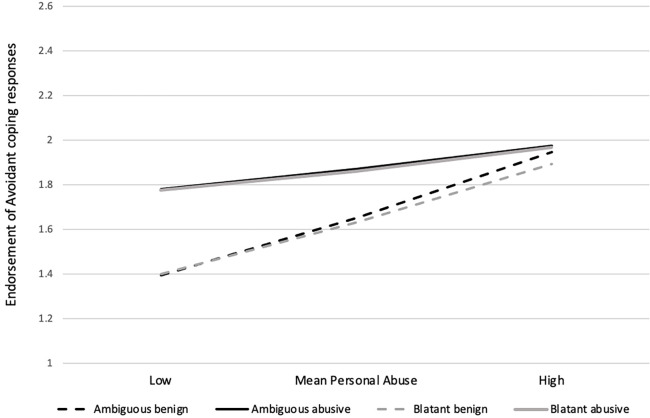
Average endorsements of avoidant coping responses to a hypothetical abusive interaction as a function of participants’ personal experiences of psychological abuse (controlling for depressive affect) and the extent to which they appraised ambiguous and blatant conflict scenarios as constituting abuse (appraised as benign was at 1 SD below the mean vs. appraisals of abusive at 1 SD above the mean) in Study 2.

In contrast, the greater propensity for those who experienced psychological abuse to endorse avoidant coping strategies (*B* = 0.21, SE = 0.05, *p* < 0.001) was mediated by the appraisals of the relationship conflict, total mediation effect = 0.10, SE = 0.03, CI_.95_ [0.04, 0.17], and in particular, appraisals of whether the conflict constituted abuse. However, the test of the interactions between participants’ personal abuse experiences and perceptions of whether the conflict was appraised as abusive was found to be significant [ambiguous conflicts, *F*(1,428) = 15.21, *p* < 0.001; blatant conflicts, *F*(1,428) = 19.63, *p* < 0.001] in relation to endorsing avoidant coping strategies. Follow-up simple effects analyses suggest that reports of more severe personal experiences of abuse were associated with a greater likelihood of endorsing avoidant coping strategies (controlling depressive affect), irrespective of the conflict appraisals. Among those whose own relationships were characterized by low levels of abuse, greater recognition of relationship conflicts as being abusive was associated with endorsing avoidant coping methods in response to such conflicts (see Figure 1). Neither participants’ gender nor the length of time that they had currently been in a relationship was a significant moderator of any of the mediated models.

## 4 General discussion

As already noted, the incidence of abusive experiences have been frequently reported, even at the dating stage of a relationship (Scherer et al., 2016; Ybarra et al., 2016). Psychological abuse, in particular, is often ambiguous, making it difficult for victims to identify their experiences as abusive (Spadine et al., 2020). Indeed, in some instances, those who are being psychologically victimized may initially be unaware of (or confused about) their predicament (Minto et al., 2021). Moreover, the people they turn to for support may be equally uncertain and unwilling to label behaviors as abusive without a better understanding of the situation (Sikström and Dahl, 2023). Victims may have difficulty communicating the severity of their situation to others, and women who had *not* been in an abusive relationship were more likely to minimize psychological abuse described by others (Hammock et al., 2015; Hall et al., 2023). The hesitancy in appraising conflicts as abusive has implications for how individuals respond, and the well-being of the person being targeted.

The present investigation assessed how relationship conflicts depicted in scenarios that reflected different forms of psychological abuse were appraised. On the whole, individuals were reluctant to interpret conflict behaviors as being abusive. As expected, this reluctance was particularly likely among those who did not report experiencing abuse in their own relationships. The appraisals of conflict behaviors were associated with the patterns of coping that were deemed appropriate. Specifically, all of the appraisal dimensions were strongly associated with emotional avoidance coping strategies (blame, containment, passivity, and withdrawal), mirroring the pattern of associations frequently observed in conjunction with depressive affect. In essence, the appraisals of relationship conflicts might be a part of the constellation of responses that prompt individuals who are experiencing abuse to engage in avoidant coping behaviors, which might serve to diminish distress (Russo and Borelli, 2023).

These findings are congruent with reports that women who reported relatively high levels of psychological abuse expressed less negative attitudes toward interpersonal violence, and tended to adopt low levels of active coping efforts and instead engaged in avoidant coping strategies (Shepherd-McMullen et al., 2015). This said, even those who did not experience abuse in their personal relationships were more likely to endorse avoidant strategies when their appraisals reflected a sensitivity to the abuse cues. Thus, having an understanding of the behaviors that constitute abuse may be critical in endorsing coping strategies that might otherwise appear to be ineffectual. For example, it has been noted that some women reported forgiving their abuser, which was accompanied by lower levels of depressive symptoms, possibly because women were able to let go of resentment and concurrently diminish their reliance on emotion-focused coping within their current relationship (Ysseldyk et al., 2009). Curiously, although forgiveness was associated with enhanced well-being among women who had been physically abused, this was less evident if they had been psychologically abused in their previous relationships (Ysseldyk et al., 2019). These findings speak to the possibility that, much like the differential perceptions of ambiguous and blatant abusive interactions in the present study, women perceived the impact of different forms of abuse differently and perhaps psychological abuse was more injurious. The impacts of such variations in the nature of the interactions, including the public versus private expressions of abuse merit further consideration.

Although the seriousness of conflicts and the distress they created were related to appraisals of ambiguous situations as abusive, surprisingly, this was not evident in regard to blatant conflicts. On the contrary, such threat appraisals were inversely related to blatant conflicts being seen as abusive, perhaps pointing to the impacts of individuals adopting an avoidant or emotional containment coping strategy. Depressive affect did not account for the associations among personal abuse experiences, appraisals, and coping. Even after we controlled for depressive affect, those individuals who reported relatively severe personal experiences of abuse were more likely to interpret conflicts as abusive, to perceive a lack of control over their resolution, and to endorse avoidant coping strategies. Thus, although depressive affect might well be a component of the distress experienced by individuals who have encountered abuse (Mazza et al., 2021), it is not sufficient to account for their sensitivity to abuse cues or the perception that the situation is not resolvable.

In ambiguous situations, the target may question themselves and their worldview. This is the notion of “gaslighting,” and when the behaviors are labeled as such, they are recognized as a strategy endorsed by some abusers (Klein et al., 2023). Indeed, the social construction of language to convey abusive experiences may contribute to a shared understanding and recognition of such behaviors, which would be reflected in appraisals of precisely the types of situations presented in our measure employing conflict scenarios.

Several studies have noted that perceivers and targets of abuse do not always share an understanding of the severity of the situation, especially when it is ambiguous, perhaps being tied to learning through experience that such behaviors are neither excusable nor normal and can have devastating consequences (Eigenberg and Policastro, 2016; Sikström and Dahl, 2023). Moreover, when the victim self-questions their experiences, the uncertainty created further undermines the extent to which listeners understand the severity of the situation (Sikström and Dahl, 2023). Indeed, women’s narratives of abuse were frequently characterized by uncertainty, minimization, and self-blame. Although not assessed in the present study, this may reflect the consequences of the dominant discourse among traumatized women that promotes fragmented memory of abusive events, even if these had been encountered repeatedly. This may foster attitudes in which women are to blame and the seriousness of the violence is minimized. The difficulty for abused women is compounded owing to the uncertainty concerning the risks inherent in speaking of their abuse, which creates a collision of doing so while simultaneously hiding the abuse experienced (Brown, 2013).

The current measure of appraisals allowed us to assess perceptions without the overlay of narrator uncertainty to assess individual differences in interpretations of abusive conflicts (based on the Profile of Psychological Abuse). In this sense, by presenting scenarios that might “objectively” constitute abuse, we are able to explore the conditions under which they are appraised as such. This has implications for support seeking and recognizing when people are in abusive situations especially among helping professions, but even in legal contexts. In part due to the personal experiences that contribute to differing appraisals of abusive behaviors, when victims of psychological abuse seek support, they might instead encounter unsupportive reactions that minimize their experience and lead them to question their own worldviews (Song and Ingram, 2002). This may be especially pertinent to victims in abusive relationships given that they are often turned away upon seeking support, resulting in these individuals having no one to turn to, so that their abusive partner essentially becomes their only source of support. In effect, nonsupportive responses may be instrumental in keeping women in their abusive situations (Bosch and Bergen, 2006). As we have indicated previously, under these conditions their “soul mate” becomes their “cell mate” (Anisman and Matheson, 2023).

A further benefit of the measure developed for the present study was that the scenarios were not inherently gendered. The behaviors depicted could be perpetrated by or against any gender. While this allows for an exploration of the perspectives of different gender identities, it created a confound in that we could not untie the appraisals of males and females from the reactions that emanated from putting themselves into the scenario. In effect, by putting themselves in the shoes of the target in the scenarios, males were appraising abuse perpetrated against males, whereas females were considering abuse perpetrated against females (we did not have a sufficient sample size to evaluate other gender identities). Consistent with other research (Savage et al., 2017; Thomas and Hart, 2023), psychological abuse targeting females was appraised as more distressing and severe than against males. As well, males’ appraisals were less likely to differentiate ambiguous from blatantly abusive behaviors, compared to females whose patterns of response were not entirely intuitive. Females regarded blatant conflict behaviors as more distressing, and more frequent abusive personal experiences were associated with lower perceived control. However, women viewed the blatant interpersonal conflicts as more resolvable than those that were ambiguous and were less likely to define them as abusive. These differences did not emerge among males.

As much as the present findings lend themselves to a better understanding of how individuals perceive abusive experiences, the data were based on self-reports and were correlational, thus limiting the conclusions that can be drawn concerning the causal factors that favor particular attitudes toward abuse. Ultimately, to fully understand the features inherent in dealing effectively with dating abuse and other forms of interpersonal violence, it may be necessary to conduct prospective studies that assess attitudes and behaviors as the dynamics of abusive situations play out. Furthermore, data were not collected regarding conflicts that did not involve abuse, thus we could not determine whether the attitudes and actions expressed were specific to abusive situations or were more broadly related to a wide array of conflicts. These situations were based on dimensions identified in a measure that was originally developed to assess psychological violence in domestic relationships, which might also have resulted in gaps in the more subtle forms of abuse that may occur in a dating situation, and especially markers of abuse that might emerge early in the relationship. Finally, individuals in the present investigation responded to the question of what they would do if they encountered such experiences. As much as this might be informative, it has long been known that what people intend to do and the actions they actually take may not be in alignment (Nabi et al., 2002). Those who are more likely to take action maintained a greater sense of responsibility for ending interpersonal violence and expressed confidence that as a third party, they could provide help to victims (Banyard and Moynihan, 2011).

There likely is not a single approach to overcoming the psychological ramifications of partner abuse experienced by all individuals. Yet, women who overcame their trauma through posttraumatic growth accepted that they had been in an abusive relationship, acquired the ability to respond effectively to emotional triggers, and sought to associate with others who were supportive and facilitated their belief that they were no longer being controlled. More than anything they developed the attitude that “I’m a winner, not a victim” (Bryngeirsdottir and Halldorsdottir, 2022). In effect, attitudes and cognitions of the abused person are needed to achieve psychological health together with positive attitudes of those around them. In this regard, the value of third parties in helping those enmeshed in abusive relationships cannot be overstated.

## Data availability statement

The raw data supporting the conclusions of this article will be made available by the authors, without undue reservation.

## Ethics statement

The studies involving humans were approved by the Carleton University Research Ethics Board. The studies were conducted in accordance with the local legislation and institutional requirements. The participants provided their written informed consent to participate in this study.

## Author contributions

KM: Conceptualization, Formal analysis, Funding acquisition, Investigation, Methodology, Project administration, Resources, Supervision, Validation, Visualization, Writing – original draft. DW: Data curation, Formal analysis, Writing – review and editing. JL: Formal analysis, Supervision, Writing – review and editing. HA: Conceptualization, Funding acquisition, Investigation, Methodology, Project administration, Resources, Supervision, Writing – original draft.
